# Application of Indocyanine Green Enhanced Fluorescence in Esophageal Surgery: A Mini Review

**DOI:** 10.3389/fsurg.2022.961856

**Published:** 2022-07-08

**Authors:** Nicola Tamburini, Matteo Chiozza, Pio Maniscalco, Giuseppe Resta, Serafino Marino, Francesco Quarantotto, Gabriele Anania, Giorgio Cavallesco

**Affiliations:** Department of Surgery, Sant’Anna University Hospital, Ferrara, Italy

**Keywords:** esophaeal cancer, surgery, fluorescence imaging, indocianin green, indocianine green angiography, chylothorax, anastomotic leak, sentinel node

## Abstract

Despite recent technological innovations and the development of minimally invasive surgery, esophagectomy remains an operation burdened with severe postoperative complications. Fluorescence imaging, particularly using indocyanine green (ICG), offers the ability to address a number of issues faced during esophagectomy. The three main indications for the intraoperative use of ICG during esophagectomy are visualization of conduit vascular supply, allow identification of sentinel nodes and visualization of the thoracic duct. The purpose of this mini review is to present an overview of current practice in fluorescence imaging utilizing ICG during esophagectomy, as well as to demonstrate how this technology can guide lymphadenectomy and reduce surgical morbidity such as anastomotic leaking and chylothorax.

## Introduction

In recent years, innovations in technique and technology have tried to reduce morbidity related to esophagectomy while improving oncologic results. Most significantly, the development of minimally invasive esophagectomy and the use of robotic surgical technology have provided the opportunity to enhance both perioperative and long-term outcomes. Despite these advancements, numerous persistent issues in esophageal resection remain unsolved, including the evaluation of conduit perfusion in preventing anastomotic leaks, proper lymphadenectomy, and thoracic duct injuries. Intraoperative fluorescence imaging (FI) is a new technique that may be able to overcome all these issues. FI consists of injecting a small amount of fluorescent dye into the patient at a certain moment throughout the procedure. Typically, a dedicated camera with a separate FI light source and sensor is employed. The FI light source generates light at a wavelength that is tailored to stimulate the fluorescent dye, which then emits light at a known wavelength back to the FI sensor. The fluorescence picture may be seen on its own or overlaid on a typical laparoscopic/thoracoscopic image, showing organ perfusion and regions of critical anatomy such as blood supply and lymphatic drainage in real time. Indocyanine green is the most often used dye in esophageal surgery. Fluorescein was previously employed in surgical applications due to the ability to view the produced fluorescence with the naked eye and without the usage of a specialized camera. ICG, on the other hand, has the benefit of greater vision of deeper tissues than fluorescein and has recently gained popularity. ICG is safe and has a long history of usage in ophthalmology, heart and vascular surgery, cosmetic and reconstructive surgery, oncologic surgery, and cardiology.

The aim of this mini-review was to analyze in the literature the comparison between the different schools of thought or controversies, the gaps in research and the future prospects of the use of ICG technology in the course of esophagectomy. In particular, we tried to define its role in the evaluation of the gastric conduit perfusion, lymph node mapping and prevention of injuries of the thoracic duct.

## Assessment of Gastric Conduit Perfusion

Despite major advancements in surgical procedures and postoperative care, anastomotic leakage after esophagectomy remains a common and sometimes lethal complication (1). Although several variables contribute to the development of anastomotic leakage, blood perfusion is considered to be one of the most significant. Because the supply of the stomach tube is delivered by a single artery, it is critical that the distal tube has to be perfused all around. In fact, the distal portion of the stomach has been shown to have reduced perfusion after dissection of the gastrocolic ligament and the transformation of the stomach into a gastric tube (2, 3). Most surgeons have always assessed the blood circulation of the gastrointestinal tract subjectively. It is generally based on a visual examination of the stomach color, artery pulsations, and bleeding from the incision edge. However, a prior study found that clinical risk assessment by surgeons had a modest predictive value for anastomotic leaking during gastrointestinal surgery (4). There is a lack of a suitable non-invasive, conveniently accessible, cost-effective, objective, and reproducible approach for assessing the viability of the anastomotic site. Optical imaging, which is based on the interaction of light and tissue, has the ability to visualize perfusion changes in real time and at high resolution. These optical imaging properties provide the capacity to analyze perfusion intraoperatively and quantitatively (5, 6). Not all the approaches have enough specificity for patient outcomes and are currently unable to objectively quantify perfusion for clinical use. The wide variety of imaging systems available, as well as the variability of quantitative parameters and perfusion endpoints, make it difficult to compare procedures in the literature. As a result, there is no evidence on which optical imaging approach and corresponding quantitative measure is more predictive of poor perfusion and patient outcomes (7). Laser doppler flowmetry (LDF) has been used to quantify organ blood perfusion. The approach, which is based on measuring Doppler frequency variations in laser light reflected by moving red blood cells, has been verified against alternative ways of assessing tissue blood flow. However, because of its intrinsic methodologic limitations, the procedure has not gained widespread use in clinical practice (8, 9). Another technique, Laser speckle contrast imaging (LSCI), which can assess tissue perfusion both visually and quantitatively, has been used to assess gastric artery blood flow. In comparison to LDF, LSCI has been shown to be less reliable in predicting actual perfusion changes, particularly speed change. In addition, the accuracy of these instruments in assessing tissue blood flow has to be confirmed (10, 11). The use of ICG for gastric perfusion evaluation prior to anastomosis has been described as a safe method in several studies (12–15). FI dye can be administered intravenously during the conduit preparation phase of the procedure and/or before completing the anastomosis in the chest or neck. In feasibility studies, Fluorescence angiography gives both qualitative and quantitative information on the health of the gastric conduit’s micro- and macro circulatory systems (13). The total perfusion may be monitored using this approach, and an optimum location for the anastomosis can be chosen by examining the speed with which the conduit is supplied as well as any regions of demarcation in the conduit. Furthermore, additional resection of the inadequately perfused gastric conduit might ensure better vascularization. There have been no randomized controlled trials on the role of ICG fluorescence imaging in preventing anastomotic leakage, however comprehensive reviews indicate that near infrared fluorescence imaging during surgery has the potential to predict anastomotic leakage (12, 14, 15). Campbell et al. demonstrated that the rate of anastomotic leakage dropped from 20% to 0% after using intraoperative vascular assessment of the gastric conduit with ICG (16). Similarly, several authors showed that intraoperative ICG fluorescence in the gastric conduit wall was a good technique for predicting the probability of anastomotic leakage (17–22). Additionally, ICG has been used to aid in the identification of the path of the right gastroepiploic artery in order to better protect it during greater omentum division (23), as well as to visualize the intramural blood supply of gastric, colonic, and jejunal conduits (13), and the vascularity of omental flaps used for anastomotic reinforcement (24).

The lack of a validated measuring method for the fluorescence signal is one of the main challenges that must be solved before assessing the real impact of this technology on anastomotic complications. In fact, there have been few studies attempting to assess the indocyanine green angiography signal during esophageal surgery based on the speed and/or intensity of the fluorescent coloring of the gastric tube following dye injection. Quantitative assessment of ICG fluorescence was introduced in the more recent studies. There are two main methods for quantification: those for assessing the intensity (25) and those for assessing the perfusion time or speed of ICG fluorescence (26). Ishige et al. (26) aimed to quantitatively assess ICG fluorescence in esophageal cancer surgery using “ROIs”, a software program that quantified the fluorescence intensity and created a time-fluorescence intensity curve to assess the blood perfusion three times intraoperatively. They showed a decline in the blood perfusion around the anastomotic site of the prepared gastric conduit. However, it is very difficult to evaluate the blood flow by administering ICG at multiple different sites in each phase. Another gap in this field regards the dose of ICG. It usually ranges between 1.25 and 25 mg for each bolus. The lowest dose permits a reasonable and solid visualization, but is not enough in some cases. However, a high dose could disrupt a subsequent estimation. If the signal is not clear, an additional bolus of 2.5 mg of ICG can be injected and after 15 min another estimation is possible (12). Additionally, although ICG fluorescent imaging is helpful in the assessment of arterial blood flow, it is difficult to evaluate venous drainage. That is a critical etiologic variable related to early (leakage) and late (stenosis) anastomotic complications (22).

Surely a future perspective in the topic will be the standardization of different data. Currently the available imaging systems have different sign responsiveness because of contrasts in hardware, optics and image processing, which makes it difficult to correlate the results. Calibration of the imaging systems could contribute to having data from different near-infrared imaging systems comparable. In future studies, artificial intelligence (AI) could work on the read-out of fluorescence angiography. Potentially AI will decrease interobserver variety and better approaches to interpret the near-infrared images in order to predict anastomotic leak.

## Sentinel Node Mapping

Sentinel nodes (SN) are described as the primary tumor’s first draining lymph nodes and, as such, the first potential locations of micrometastasis along the lymphatic drainage route from the primary tumor site. A histopathological examination of the SN is thought to be effective in determining the extent of lymph node metastases. Standard lymphadenectomy can be avoided when SN are found and proven to be cancer-free. The role of sentinel lymph node (SLN) mapping in cancer prognosis was initially outlined more than a half century ago and has since been included into the normal care of numerous solid tumors such as melanoma and breast cancer (27–30). Esophageal cancer is characterized with a complex pattern of lymph node metastases that extends from the cervical to the abdominal area. Lymph node metastasis is not a rare event in esophageal cancer, and the incidence of lymph node metastasis, even in pT1b tumors, reaches 45% (31). Lymph node status is the absolute most significant prognostic factor, deciding the area of the primary cancer and the lymphatic spread of the disease, which can establish the ideal therapeutic strategy for a patient (32). Using lymphatic mapping, surgeons might avoid leaving lymph nodes that are likely to carry metastases unresected and instead perform a targeted lymphadenectomy to improve staging. Indocyanine-green (ICG) imaging is a safe and simple strategy for sentinel lymph node mapping (SLNM) and has been widely used in patients going through malignant surgery. Near-infrared systems with fluorescence colorants (ICG), have been shown to be a helpful method for the discovery of sentinel lymph nodes in different types of cancer (33). Some studies reported the feasibility of NIR image-guided lymphatic mapping in patients with esophageal cancer undergoing surgery with a good detection rate (34–36).

To date, the main gaps in research in this field concern the low sample size in the studies, especially with regard to certain variables such as the association between detection rate and preoperative chemoradiotherapy. The lowest detection rate was seen in patients with neoadjuvant chemoradiotherapy (54%), but only three studies explicitly detailed the post-chemoradiotherapy location rate (37–49). Another challenge is the interval between injection and intervention; because few studies take this into account, this information cannot be extended to the full population, and further research is needed to address this

The potential future and potential developments of the use of ICG in lymph node mapping during esophagectomy mainly concerns the standardization of this technique during intervention. This approach has now been proven to be safe, but further research is needed before it can be used on a regular basis.

## Prevention of Thoracic Duct Injuries

Chylothorax is an infrequent but relatively serious complication after general thoracic surgery associated with potentially life-threatening respiratory and metabolic morbidity (40). Chylothorax after esophageal surgery occurs in 2%–12% of cases. It is a complication associated with increased hospital stay and mortality: in fact studies reported an increased risk of malnutrition, respiratory infection, respiratory failure and sepsis in up to 24% of cases. Because of the thoracic duct’s (TD) varied course in the thoracic cavity, esophageal surgery has a significant risk of causing injury to the TD and its branches. Furthermore, TD is frequently not detectable during surgery, especially in obese patients, making lesion prevention and repair challenging. Although non-operative measures, such as the complete cessation of oral intake and pleurodesis, are effective treatment methods for chylothorax complicating pulmonary resection (41), early surgical intervention should be performed in high-output chylous leakage patients (42). However, it could be very difficult to repair the TD leakage, especially during re-intervention, and usually, mass ligation of the TD is performed instead.

**Figure 1:**
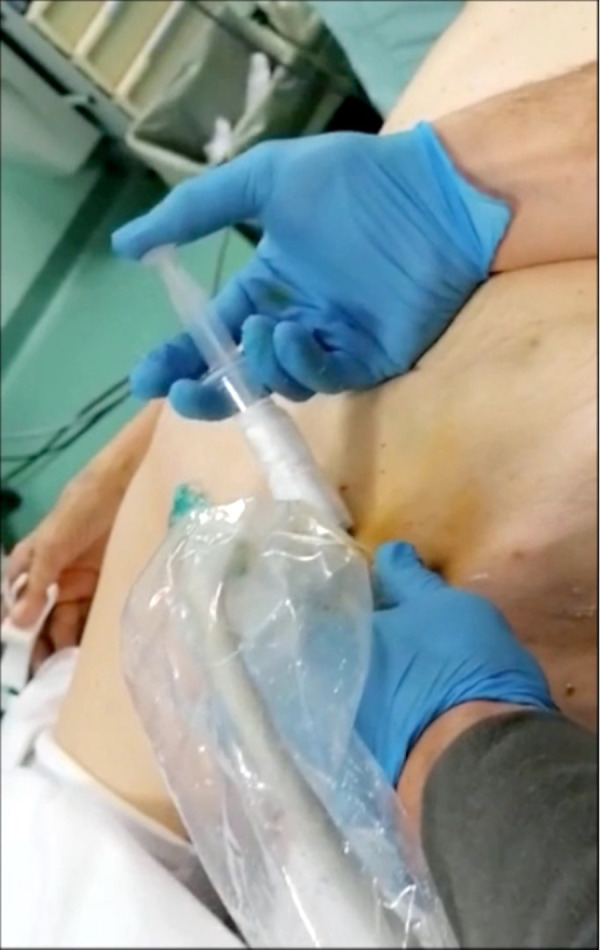
A dose 0.5 mg/kg of ICG is injected percutaneously bilaterally in the superficial inguinal nodes under ultrasound visualization. This procedure is performed before thoracoscopy in total esophagectomy, or after laparoscopic time in Ivor Lewis esophagectomy.

**Figure 2:**
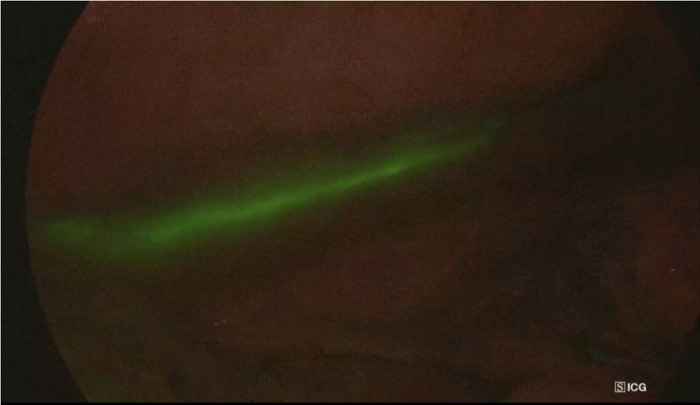
Intraoperative NIR imaging of thoracic duct in a patient with diagnosed distal oesophageal cancer. The procedure allowed surgeons to identify the thoracic duct during surgery.

There have been several strategies reported for visualizing chylous effusion. Lymphoscintigraphy using technetium-99 (Te-99) imaging (43) or magnetic resonance-thoracic ductography (44) could not properly and intraoperatively define the damaged site. Preoperative fat meal administration through mouth or nasogastric tube has been shown to improve TD visibility and reduce iatrogenic duct damage (45). However, the reproducibility of this procedure is weak. Only a few studies in humans have been published on NIR imaging of the TD in patients of chylothorax (46–50). The approach has been described as an effective method for identifying the TD because it provided a clear anatomic view of the TD, as well as the tributary and aberrant branches (Figures 1, 2). The surgeon’s dissection is safe because of the continual control of the anatomy and the possibility to switch images from ordinary light to NIR mode. Furthermore, this technique can be used to define the exact site of a fistula in case of TD lesion.

The standardization of the procedure is one of the major concerns. In fact the thoracic duct visualization varied across the studies between 5 and 52 min after subcutaneous injection at bilateral inguinal region or into the mesentery of the small bowel (46–50). More cases are needed to determine with certainty if the site of injection and the concentration used may be standardized. Given the paucity of data in literature at the moment it is uncertain if this tailored method is more effective than typical mass ligation based on anatomic features.

## Conclusion

Fluorescence imaging with ICG is a promising and safe method for reducing surgical morbidity following esophageal resection with continuity restoration. Anastomotic leakage was reduced using ICG fluorescence angiography. Future studies are needed to establish the feasibility of ICG lymphography for preventing chyle fistulas and to guide lymphadenectomy.
